# Supplementary material for: Adverse Events and Associated Economic Burden of COVID-19 Vaccination in Queensland, Australia: Findings from the Cross-Sectional QoVAX-Statewide Study
Source: Vaccines (Basel). 2025 Jun 30;13(7):712. doi: 10.3390/vaccines13070712 (PMC12300968; doi:10.3390/vaccines13070712)

Supplementary Table S1. Existing evidence investigate the broader impact of COVID-19 adverse events in Australian setting.

Author	Year	Location	Study design	Data source	Target population	Vaccine dose	Sample size	Age	Female	AEFIs	Medical care/advice due to AEFIs	Absence from work	Economic analysis
**Deng**	2022	All Australian states	Prospective observational cohort	AusVaxSafety surveillance	People aged 16 years or more who received COVID-19 vaccines during 22 February to 30 August 2021 at one of the vaccination sites monitored by AusVaxSafety	first and second dose	3,035,983	The median age of Comirnaty recipients (dose 1, 42 [IQR 33–49] years; dose 2, 44 [IQR 37–49] years) was lower than that of Vaxzevria recipients (dose 1, 61 [IQR 52–68] years; dose 2, 62 [IQR, 54–70]).	56.7%	35.9% of respondents reported AEFI 0–3 days after Comirnaty dose 1, 54.7% after Comirnaty dose 2, 52.8% after Vaxzevria dose 1, and 22.0% after Vaxzevria dose 2. Local pain, fatigue, headache, and myalgia were the most frequently reported symptoms.	28,298 respondents (0.9%) sought medical review in the three days following vaccination, most frequently following Comirnaty dose 2 (1.4%) or Vaxzevria dose 1 (1.2%); 5,107 people (0.2%) reported emergency department presentations	Comirnaty dose 1, 6.8% reported missed work, study or routine activities, 20.3% after Comirnaty dose 2, 17.4% after Vaxzevria dose 1, and 4.2% after Vaxzevria dose 2.	No
**Hamilton**	2022	Queensland	Prospective observational cohort	AusVaxSafety surveillance	all adults aged ≥16 years presenting for their second dose of COVID-19 vaccination at the Hervey Bay COVID-19 Vaccination Clinic	second dose	1,148	Most participants were aged between 50–70 years (55.2%)	61.3%	AEFIs were reported in 514 (44.8%) participants. Reported AEFI most frequently occurred on Day 1–3 post vaccine, followed by the day of vaccination.	Medical assistance sought 16 (1.4% for the overall cohort)	Missed work, study or routine activities 77 (6.7% for the overall cohort); the median days missed was 2 (IQR 1-3)	No

Supplementary Table S2. Project Plan for the economic analysis of QoVAX Statewide Study.

Economic burden associated with adverse events due to COVID-19 vaccination

Project Plan

(*Metro North Health owns the copyright on this document*)

1. Introduction

1.1 Purpose

This program of work will overview the epidemiological approach to the analysis of the QoVAX SET Pilot data. Data will be collected and stored by other providers and teams, the purpose of this document it to overview the plan of analysis for the collected data and the presentation of the results.

2. Project Overview

2.1 Project Description

The rollout of the COVID-19 vaccination program across Queensland presents the challenge of evaluation of the safety and efficacy of the program in our community. The Queensland COVID-19 Vaccination Safety and Efficacy Trial (QoVax SET) Program is effectively a Phase 4 study focused on preventative health benefits of the vaccine for selected participant groups as a representation of the entire Queensland population.

Recipients of the COVID-19 vaccination in Queensland will be invited to participate in a population-based study of the vaccine safety and effectiveness, linking longer-term vaccine outcome analysis to immunological and phenotypic markers. Without appreciable prevalence of SARS-CoV-2 in our community, vaccine efficacy can only be inferred by surrogate early indicators of efficacy based on induction of immune (antibody and cellular) markers of likely protection from infection, transmission, and/or severe disease outcomes. This study will focus on the induction and function of specific humoral and cellular immunity to SARS-CoV-2, and the longevity of sustained specific antibody and cell mediated immunity, in different subsections of the Queensland population and to gauge herd immunity of the population. The study sets up a unique long-term opportunity to identify and pursue better health and socioeconomic outcomes related to COVID-19 testing and vaccination regimes, support broader health and wellbeing outcomes for our state, and to accelerate further related translational research activities.

Such information will directly influence government decisions on when it is safe to open borders and resume full national and international engagement. These decisions will have profound health, social and economic impact on our community.

Further information regarding the program protocol can be found in Attachment 1.

2.2 Objectives

The overarching aims of the QoVax SET Pilot are to investigate the safety and efficacy of the COVID-19 vaccine(s) in Queensland, examine host and intrinsic factors that influence induction of immune responses to the vaccine, and to help reduce the overall burden of COVID-19 on the Queensland community including vulnerable people and the health system.

The primary research question for the QoVax SET study is:

> Across sub-sections of the Queensland community, are the COVID-19 vaccines (Pfizer, AstraZeneca or Novavax) administered by Queensland Health safe and efficacious as indicated by early induction of (humoral, antibody and cellular) immune biomarkers at one-month post second dose?

Additional secondary research questions have been posed that this project can address including:

- Is the vaccine safe for all in our community, based on questionnaire responses and routine reporting of adverse events?
- For how long, and in which groups of the population, will vaccination offer protection from severe disease by community acquired SARS-CoV-2 infection? (when borders open)?
- What is the incidence of medium- to long-term unreported severe adverse events due to the vaccine e.g. hospitalisations linked to COVID-19 vaccination, emergency department visits, indicated by surveillance of Queensland Health clinical information systems?
- Are there unanticipated and emerging effects of the vaccine, indicated by continuous surveillance of the Queensland Health clinical information systems for individuals consenting to take part?
- Will all vulnerable groups (e.g. Aboriginal and Torres Strait Islanders, refugee communities, elderly, immunocompromised) who are at risk of SARS-CoV-2 infection and disease, have equitable access, acceptance and response to the vaccine?
- What are the host intrinsic (immunological T and B cell receptor repertoires) and extrinsic factors (socio-economic health determinants, environmental exposures e.g. smoking) associated with response variation, measured by indicators of immunity following vaccination?
- Does prior infection with SARS-CoV-2 or (other coronaviruses; e.g. NL63, OC43, 299E) affect induction of immunity to SARS-CoV-2 by the vaccines?
- Do certain pre-existing health conditions (autoimmunity, cancer, immunodeficiency), and medication use effect vaccine efficacy?
- Is Pfizer or AstraZeneca more effective in the Queensland community (in recipients matched for demographic and health status)?

The objectives of this study are to:

1) Provide a guidance on the methods used for data analysis of the QoVAX project,
2) Define the relationships between demographic, social and medical-health determinants of COVID-19 vaccination outcomes and,

3) Provide high-level epidemiological analysis to the collected QoVAX dataset to understand the determinants of successful vaccination program.

2.3 Methods

The overall methods for the QoVAX SET Pilot and Mixed Dose studies are detailed are described in the respective project protocols which underpin this sub-study.

The aims for this sub-study are outlined below:

Primary aims: For participants enrolled in the QoVax pilot (including Pilot Boost participants) and QoVax Mixed dose studies:

- To investigate the direct healthcare resource use and costs of vaccination-related adverse events
- To quantify the indirect (productivity) costs from lost productivity due to experiencing vaccination-related side effects.
- To investigate whether the **cost burden differs between participant subgroups** based on their sociodemographic and clinical characteristics.
- To identify key cost drivers to inform future policy and planning.

Research questions:

This study will address these specific research questions:

- What is the scale and profile of the healthcare costs associated with COVID-19 vaccination adverse events, including ED presentations and unplanned admissions?
- What are the indirect (productivity) costs associated with COVID-19 vaccination-related side effects?
- Do direct healthcare costs and indirect costs associated with COVID-19 vaccination-related side effects differ by the type of vaccination administered and/or sociodemographic and other characteristics of participants?
- What are the key drivers of costs in relation to the COVID-19 vaccination-related side effects and adverse events?
- What are the clinical, budgetary and healthcare policy impactions of these findings?

Data items

De-identified data from participants enrolled in the QoVax Pilot and Mixed Dose studies will be required. Specifically, the following range of data items will be included in the analysis:

Baseline	Post-survey
• Record ID<br>• Vaccine site<br>• Vaccine date<br>• Age<br>• Gender	• Vaccine type<br>• Time off work (Pilot study only)<br>• Time off other activities (Pilot study only)<br>• Side effects

• Aboriginal and Torres Strait Islander status<br>• Education level<br>• Employment status<br>• Occupation<br>• Income range<br>• Chronic conditions<br>• Smoking status<br>• General wellbeing scores<br>• COVID-19 status and severity	• Linked health service resource use data: hospital admissions, ED presentations, non-admitted patient data

Analysis

The Queensland Health system perspective will be used to analyse the direct healthcare costs in relation to COVID-19 vaccination-related side effects. The indirect (productivity) costs will be estimated from the participant perspective and include absenteeism due to the management of vaccination-related side-effects. Average-per-participant (direct, indirect, and overall) costs in relation to vaccination-related side-effects will be estimated. Cost data will be described as mean ± SD and 95% confidence intervals. Costs will be disaggregated across ED, hospital and non-admitted patient settings and all costs will be presented in 2022 Australian dollars. Costs will be reported for the overall sample and by patients' subgroups based on their vaccination type and socioeconomic, demographic and other features. Univariable and multivariable regression models will be used to identify the key drivers of economic burden of COVID-19 Vaccination-related side effects.

Outcomes:

- Adverse event-related resource use and costs per participant
- Indirect (productivity) costs due to vaccination related side effects per participant
- Key drivers of costs associated with vaccination related side effects and adverse events

Research outputs: one peer-reviewed journal article plus one national conference presentation.

Supplementary Table S3. Chronic conditions

Rank	Chronic conditions	Yes	No	%
1	Other health conditions	1375	5422	20.23
2	Allergic diseases	748	6049	11.00
3	Chronic lung disease	457	6340	6.72
4	Diabetes	340	6457	5.00
5	Chronic inflammatory condition	287	6510	4.22
6	Autoimmunity	246	6551	3.62
7	Heart disease	235	6562	3.46
8	Poorly controlled blood pressure	169	6628	2.49
9	History of anaphylaxis	161	6636	2.37
10	Cancer	106	6691	1.56
11	Neurological condition	74	6723	1.09
12	Immunosuppressed	74	6723	1.09
13	Chronic kidney disease	47	6750	0.69
14	Blood cancer	39	6758	0.57
15	Previous adverse vaccine response	33	6764	0.49
16	Chronic liver disease	30	6767	0.44
17	Immunodeficiency	23	6774	0.34
18	Currently receiving treatment for cancer	16	6781	0.24
19	Organ transplant recipient	9	6788	0.13
20	On dialysis	2	6795	0.03
21	Bone marrow transplant recipient	0	6797	0.00

Supplementary Table S4. Participants' characteristics at the time of survey (Each dose).

	Dose 1 (N = 6777)			Dose 2 (N = 6747)			Dose 3 (N = 6268)			Dose 4 (N = 3084)		
**Variable**		**Value**	**%**		**Value**	**%**		**Value**	**%**		**Value**	**%**
**Age**, years	n=6777			n=6747			n=6268			n=3084		
Mean (SD)		53.1 (14.5)	NA		53.1 (14.5)	NA		53.8 (14.4)	NA		60.5 (12.4)	NA
Median		54.2 (42.2, 64.0)	NA		54.2 (42.2, 64.1)	NA		55.0 (43.2, 64.6)	NA		62.1 (52.3, 69.8)	NA
Min, Max		[18.0, 96.5]	NA		[18.0, 96.5]	NA		[18.0, 96.5]	NA		[20.67, 96.5]	NA
**Gender**	n=6777			n=6747			n=6268			n=3084		
Male		2179	32.1%		2167	32.1%		2034	32.5%		1133	36.7%
Female		4590	67.5%		4572	67.8%		4228	67.5%		1950	63.2%
Missing		6			6			5			1	
**Aboriginal and/or Torres Strait Islander**	n=6711			n=6681			n=6214			n=3068		
Yes		147	2.2%		146	2.2%		113	1.8%		48	1.6%
No		6513	97.0%		6485	97.1%		6059	97.5%		3000	97.8%
Missing		51	0.8%		50	0.7%		42	0.7%		20	0.7%
**BMI**, years	n=6621			n=6591			n=6130			n=3032		
Mean (SD)		28.0 (6.3)	NA		28.0 (6.3)	NA		28.0 (6.3)	NA		28.1 (6.2)	NA
Median		26.9 (23.7, 31.2)	NA		26.9 (23.7, 31.2)	NA		26.9 (23.7, 31.2)	NA		27.1 (24.0, 31.2)	NA
Min, Max		[15.4, 94.3]	NA		[15.4, 94.3]	NA		[15.4, 94.3]	NA		[15.4, 94.3]	NA
**Obesity**	n=6621			n=6591			n=6130			n=3032		
Yes (BMI >30 kg/m^2)		2051	31.0%		2046	31.0%		1902	31.0%		958	31.6%
No		4570	69.0%		4545	69.0%		4228	69.0%		2074	68.4%
**Education**	n=6707			n=6677			n=6212			n=3068		
High school or below		1202	17.9%		1200	18.0%		1104	17.8%		623	20.3%
Certificate/diploma degree		1879	28.0%		1864	27.9%		1701	27.4%		847	27.6%
Bachelor degree		1638	24.4%		1635	24.5%		1522	24.5%		655	21.3%
Postgraduates or above		735	11.0%		732	11.0%		691	11.1%		339	11.0%
Missing		1253	18.7%		1247	18.7%		1194	19.2%		604	19.7%
**Labour force status**	n=6078			n=6678			n=6212			n=3068		
Full time		3019	49.7%		3004	45.0%		2769	44.6%		1048	34.2%
Part time		1204	19.8%		1198	17.9%		1112	17.9%		463	15.1%
Casual		420	6.9%		442	6.6%		374	6.0%		154	5.0%
Unemployed		160	2.6%		60	0.9%		139	2.2%		75	2.4%
Not in labour force (this includes carers, volunteers, students, retirees, and home duties)		183	3.0%		181	2.7%		169	2.7%		110	3.6%
Other		1696	27.9%		1686	25.2%		1626	26.2%		1026	33.4%
Missing		26	0.4%		27	0.4%		23	0.4%		12	0.4%
**Chronic conditions**	n=6777			n=6747			n=6268			n=3084		
0		4053	59.8%		4037	59.8%		3725	59.4%		1650	53.5%
1		1592	23.5%		581	8.6%		1481	23.6%		811	26.3%
>1		1132	16.7%		1129	16.7%		1062	16.9%		623	20.2%
**EQ-5D-5L**	n=6603			n=6575			n=6111			n=3026		
Mean (SD)		0.94 (0.11)	NA		0.94 (0.11)	NA		0.94 (0.10)	NA		0.94 (0.10)	NA
Median		0.96 (0.92, 1.00)	NA		0.96 (0.92, 1.00)	NA		0.96 (0.92, 1.00)	NA		0.96 (0.92, 1.00)	NA
**Vaccine type**	n=6777			n=6747			n=6268			n=3084		
BNT 162b2 mRNA vaccine		4269	63.0%		4295	63.7%		5568	88.8%		2172	70.4%
ChAdOx1 adenoviral vector		2472	36.5%		2389	35.4%		44	0.7%		15	0.5%
mRNA-1273		14	0.2%		26	0.4%		596	9.5%		781	25.3%
NVX-CoV2373		2	0.0%		5	0.1%		16	0.3%		45	1.5%
Not sure		20	0.3%		32	0.5%		44	0.7%		71	2.3%
**Adverse effect**	n=6777			n=6747			n=6268			n=3084		
Yes		3617	53.4%		2978	44.1%		2550	40.7%		1262	40.9%
No		2968	43.8%		3548	52.6%		3525	56.2%		1747	56.6%
Missing		192	2.8%		221	3.3%		193	3.1%		75	2.4%

ABS definition for full-time, part-time, casual, and unemployment work: https://www.abs.gov.au/statistics/detailed-methodology-information/concepts-sources-methods/labour-statistics-concepts-sources-and-methods/2023/concepts-and-sources/employment-arrangements#status-in-employment.

Abbreviations: BMI, body mass index; SD, standard deviation; NA, not applicable; EQ-5D-5L, EuroQol five-dimension five-level.

Supplementary Table S5. Number of participants reporting AEFIs among each dose, by AEFI type (local and systemic).

		Dose 1		Dose 2		Dose 3		Dose 4	
		**Number**	**%**	**Number**	**%**	**Number**	**%**	**Number**	**%**
**Local AEFIs**	No AEFIs	463	6.8%	335	5.0%	272	4.3%	113	3.7%
	Number of AEFIs	3154	46.5%	2643	39.2%	2278	36.3%	1149	37.3%
	1	*771*	*11.4%*	*748*	*11.1%*	*665*	*10.6%*	*374*	*12.1%*
	2	*875*	*12.9%*	*714*	*10.6%*	*604*	*9.6%*	*314*	*10.2%*
	3	*633*	*9.3%*	*461*	*6.8%*	*390*	*6.2%*	*197*	*6.4%*
	4	*655*	*9.7%*	*512*	*7.6%*	*446*	*7.1%*	*202*	*6.5%*
	5	*220*	*3.2%*	*208*	*3.1%*	*173*	*2.8%*	*62*	*2.0%*
**Systemic AEFIs**	No AEFIs	721	10.6%	687	10.2%	732	11.7%	394	12.8%
	Number of AEFIs	2896	42.7%	2291	34.0%	1818	29.0%	868	28.1%
	1	*677*	*10.0%*	*520*	*7.7%*	*421*	*6.7%*	*203*	*6.6%*
	2	*564*	*8.3%*	*440*	*6.5%*	*353*	*5.6%*	*184*	*6.0%*
	3	*459*	*6.8%*	*377*	*5.6%*	*282*	*4.5%*	*148*	*4.8%*
	4	*348*	*5.1%*	*294*	*4.4%*	*239*	*3.8%*	*121*	*3.9%*
	5	*293*	*4.3%*	*208*	*3.1%*	*165*	*2.6%*	*77*	*2.5%*
	6	*219*	*3.2%*	*167*	*2.5%*	*120*	*1.9%*	*49*	*1.6%*
	7	*189*	*2.8%*	*139*	*2.1%*	*124*	*2.0%*	*54*	*1.8%*
	8	*143*	*2.1%*	*138*	*2.0%*	*111*	*1.8%*	*32*	*1.0%*
	9	*<5*	*<0.1%*	*8*	*0.1%*	*<5*	*<0.1%*	*<5*	*<0.1%*

Footnote: AEFIs, adverse effects following immunization. Local symptoms included heat, swelling, pain, itch, and tenderness; systemic symptoms included fever, Nausea, shivering, chill, headache, tiredness, muscle ache, joint pain, and clot.

Supplementary Table S6. Number of participants reporting each adverse effect, by duration.

	**Dose 1 (n=3617)**						**Dose 2 (n=2978)**						**Dose 3 (n=2550)**						**Dose 4 (n=1262)**					
	No AEFIs	*≤24 hours*	*1-3 days*	*4-7 days*	*> 1 week*	*not sure*	*No AEFIs*	*≤24 hours*	*1-3 days*	*4-7 days*	*> 1 week*	*not sure*	*No AEFIs*	*≤24 hours*	*1-3 days*	*4-7 days*	*> 1 week*	*not sure*	*No AEFIs*	*≤24 hours*	*1-3 days*	*4-7 days*	*> 1 week*	*not sure*
**Local AEFIs**																								
Heat	2080	667	614	137	35	77	1760	594	451	87	31	55	1531	472	392	86	27	41	817	195	175	53	7	15
Swelling	2239	520	588	144	49	70	1846	451	488	104	35	54	1539	399	432	106	34	40	829	156	187	76	5	9
Pain	1356	818	1099	212	79	47	1162	768	835	133	45	35	1008	630	697	148	39	28	527	269	368	81	13	*<5*
Itch	3145	167	151	55	20	72	2579	147	141	41	17	53	2218	126	109	33	17	46	1129	43	49	25	5	11
Tenderness	792	985	1385	313	102	35	673	933	1080	194	72	26	589	778	916	197	54	16	257	342	516	131	15	*<5*
**Systemic AEFIs**																								
Fever	2481	636	392	46	16	40	2117	453	310	46	18	34	1901	337	234	41	10	27	1002	138	101	7	*<5*	11
Nausea	2946	317	237	38	30	40	2431	225	216	47	27	32	2109	211	147	30	21	31	1094	96	55	7	*<5*	7
Shivering	2831	461	237	33	11	38	2428	264	219	28	11	28	2090	209	183	28	10	30	1081	97	67	7	*<5*	8
Chill	2730	533	263	33	11	40	2351	318	238	28	13	30	2037	244	201	30	10	28	1050	113	81	9	*<5*	7
Headache	1759	952	642	113	87	58	1427	775	572	94	65	45	1312	644	414	93	54	33	700	286	218	36	16	6
Tiredness	1302	1005	919	197	148	40	1094	756	784	185	129	30	1040	648	568	165	102	27	549	319	300	59	30	5
Muscle ache	1935	688	712	141	87	45	1607	531	601	117	81	41	1468	428	429	120	70	35	760	220	215	44	18	5
Joint pain	2694	300	376	109	70	62	2204	236	332	87	77	42	1944	185	239	77	66	38	993	93	118	33	18	7
Clot	3566	*<5*	*<5*	*<5*	9	27	2938	*<5*	*<5*	*<5*	7	25	2522	5	*<5*	*<5*	*<5*	18	1254	*<5*	*<5*	*<5*	*<5*	7
**Other AEFIs**																								
	3128	115	98	72	164	32	2579	68	89	61	160	21	2152	63	112	73	132	16	1138	27	35	21	32	9

Footnote: AEFIs, adverse effects following immunization.

Supplementary Table S7. Healthcare costs (AU$)

		Unit cost	Number (%) of individuals	Average number of times	Overall cost	Reference for unit cost
Dose 1	13 Health	$11.115	31	1.26	$434	the wage rates as of 2022 for Registered Nurse in Queensland Health
	Ambulance	$790	14	1.26	$13,936	cost per incident reported in the Queensland Ambulance Service 2022
	General practitioner	$129.65	135	2.48	$43,407	MBS item 5060
	Emergency department	$64.60	62	1.29	$5,167	MBS item 5001
	Community clinic nurse or Indigenous Health Worker	$26.40	6	1.00	$158	MBS item 10987
	Admitted to public hospital #	$5,797*price weigh	-	1	$2,190	AR-DRG Code X63B (price weight: 0.3778)
			-	1	$784	AR-DRG Code J68B (price weight: 0.1353)
			-	2	$13,567	AR-DRG Code F63A (price weight: 1.1702)
			-	1	$7,389	AR-DRG Code X63A (price weight: 1.2746)
	**Total cost**				**$87,032**	
	**Cost per person reporting AEFIs**				**$24**	
	**Cost per person vaccinated (overall cohort)**				**$13**	
Dose 2	13 Health	$11.115	20	2.00	$445	the wage rates as of 2022 for Registered Nurse in Queensland Health
	Ambulance	$790	18	2.00	$28,440	cost per incident reported in the Queensland Ambulance Service 2022
	General practitioner	$129.65	120	4.50	$70,011	MBS item 5060
	Emergency department	$64.60	47	1.68	$5,101	MBS item 5001
	Community clinic nurse or Indigenous Health Worker	$26.40	5	1.60	$211	MBS item 10987
	Admitted to public hospital #	$5,797*price weight	-	1	$1,379	AR-DRG Code F75B (price weight: 0.2379)
			-	2	$2,313	AR-DRG Code B77B (price weight: 0.1995)
			-	4	$7,302	AR-DRG Code F73B (price weight: 0.3149)
			-	1	$821	AR-DRG Code F74B (price weight: 0.1416)
			-	1	$682	AR-DRG Code D61A (price weight: 0.1176)
			-	11	$139,918	AR-DRG Code F42A (price weight: 2.1942)
			-	1	$2,190	AR-DRG Code X63B (price weight: 0.3778)
			-	2	$2,313	AR-DRG Code B77B (price weight: 0.1995)

	**Total cost**				**$261,125**	
	**Cost per person reporting AEFIs**				**$88**	
	**Cost per person vaccinated (overall cohort)**				**$39**	
**Dose 3**	13 Health	$11.115	12	1.17	$156	the wage rates as of 2022 for Registered Nurse in Queensland Health
	Ambulance	$790	6	1.17	$5,546	cost per incident reported in the Queensland Ambulance Service 2022
	General practitioner	$129.65	86	2.68	$29,882	MBS item 5060
	Emergency department	$64.60	21	1.38	$1,872	MBS item 5001
	Community clinic nurse or Indigenous Health Worker	$26.40	2	1.50	$79	MBS item 10987
	Admitted to public hospital #	$5,797*price weight	-	4	$18,165	AR-DRG Code E61B (price weight: 0.7834)
			-	2	$1,363	AR-DRG Code D61A (price weight: 0.1176)
	**Total cost**				**$57,064**	
	**Cost per person reporting AEFIs**				**$22**	
	**Cost per person vaccinated (overall cohort)**				**$9**	
**Dose 4**	13 Health	$11.115	2	1.00	$22	the wage rates as of 2022 for Registered Nurse in Queensland Health
	Ambulance	$790	1	1.00	$790	cost per incident reported in the Queensland Ambulance Service 2022
	General practitioner	$129.65	20	1.70	$4,408	MBS item 5060
	Emergency department	$64.60	1	1.30	$84	MBS item 5001
	Community clinic nurse or Indigenous Health Worker	$26.40	0	0.00	$0	MBS item 10987
	Admitted to public hospital #	$5,797*price weight	-	-	-	-
	**Total cost**				**$5,304**	
	**Cost per person reporting AEFIs**				**$4**	
	**Cost per person vaccinated (overall cohort)**				**$2**	

National efficient cost for 2022-23 is $5,797.

Supplementary Table S8. Absenteeism costs (AU$)

	Dose 1	Dose 2	Dose 3	Dose 4
Overall absenteeism costs#	880,233	1,277,451	432,663	95,222
Number of individuals who reported sick leaves*	589	535	381	138
Absenteeism costs per person (who reporting taking time off)				
Mean (SD)	1494 (7569)	2388 (11995)	1136 (3778)	690 (2197)
Median (IQR)	368 (316, 786)	393 (326, 786)	393 (326, 760)	355 (326, 651)
Min, Max	[51, 112441]	[89, 116791]	[116, 51381]	[35, 25953]
**Absenteeism cost per person reporting AEFI**	$1494	$2388	$1136	$690
**Absenteeism costs per person vaccinated (overall cohort)**	130	189	69	31

# Overall absenteeism costs were estimated by multiplying the number of hours absent during the study period with average wage rates, by age group (15–19, 20–24, 25–34, 35–44, 45–54, 55–59, 60–64, 65 and over), gender (males and females) and work category (full time, part time, and others). Wages were based on the 2022 Queensland Average Weekly Earnings from the Australian Bureau of Statistics (ABS).

* The number of individuals reporting sick leaves was 597, 542, 388, and 141 for Doses 1 to 4, respectively. Few participants had no complete information for the absenteeism cost estimation, and this was driven by the incomplete information on their age, gender, or work type.

Supplementary Figure S1. Percentage of participants reporting local or systemic adverse effects among each dose, by duration*.

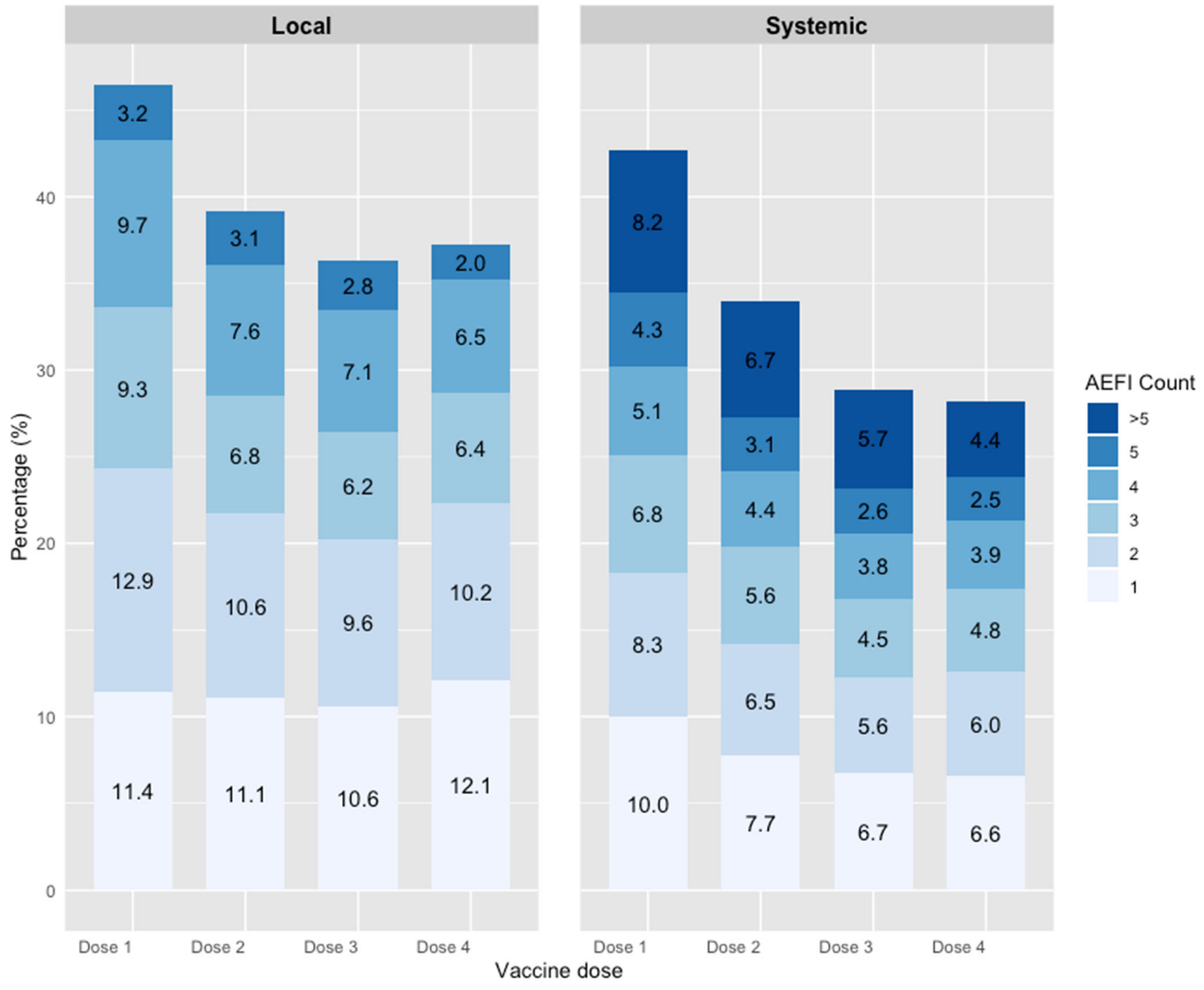

* The percentage of participants with any local adverse effects for dose 1, 2, 3, 4 was 46.5%, 39.2%, 36.3%, 37.3%, respectively, and the percentage for systemic adverse effects was 44.2%, 35.4%, 31.1%, 29.1%, respectively.

Local symptoms included heat, swelling, pain, itch, and tenderness; systemic symptoms included fever, Nausea, shivering, chill, headache, tiredness, muscle ache, joint pain, clot.

Abbreviation: AEFIs, adverse effects following immunization.

Supplementary Figure S2. Distribution of absenteeism days.

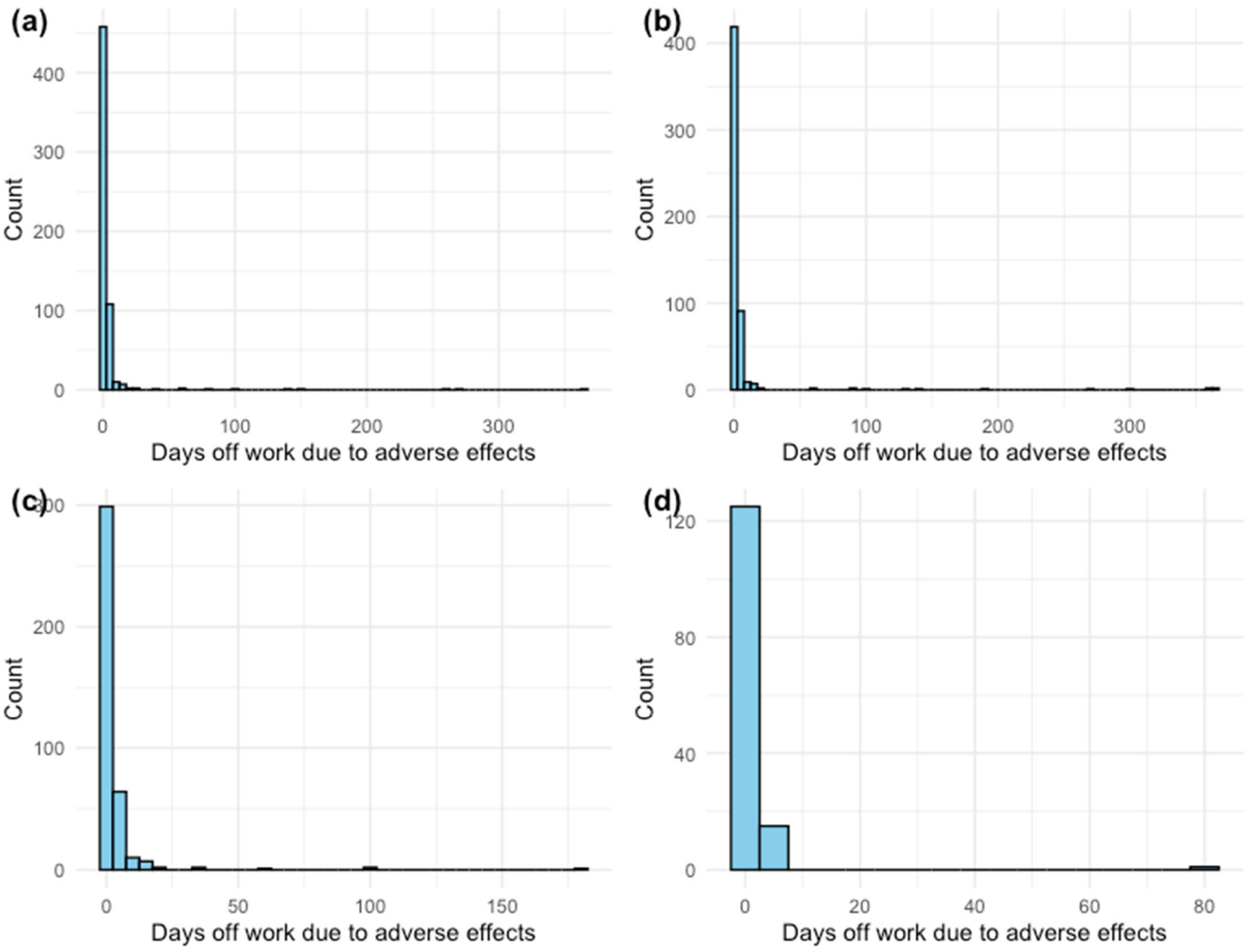

Supplement: Supplementary file 1 [file vaccines-13-00712-s001.zip › vaccines-3684807-supplementary.pdf]
